# T-cell receptor gene therapy targeting melanoma-associated antigen-A4 by silencing of endogenous TCR inhibits tumor growth in mice and human

**DOI:** 10.1038/s41419-019-1717-8

**Published:** 2019-06-17

**Authors:** Qian Sun, Xiying Zhang, Limei Wang, Xujie Gao, Yanjuan Xiong, Liang Liu, Feng Wei, Lili Yang, Xiubao Ren

**Affiliations:** 1 Department of Immunology, Key Laboratory of Cancer Immunology and Biotherapy, Key Laboratory of Cancer Prevention and Therapy, Tianjin’s Clinical Research Center for Cancer, Tianjin Medical University Cancer Institute and Hospital, Tianjin, China; 2 Department of Biotherapy, Key Laboratory of Cancer Immunology and Biotherapy, Key Laboratory of Cancer Prevention and Therapy, Tianjin’s Clinical Research Center for Cancer, Tianjin Medical University Cancer Institute and Hospital, Tianjin, China; 3 Department of Radiology, Key Laboratory of Cancer Prevention and Therapy, Tianjin’s Clinical Research Center for Cancer, Tianjin Medical University Cancer Institute and Hospital, Tianjin, China

**Keywords:** Cancer immunotherapy, Translational research

## Abstract

Genetically engineered T cells expressing a T-cell receptor (TCR) are powerful tools for cancer treatment and have shown significant clinical effects in sarcoma patients. However, mismatch of the introduced TCR α/β chains with endogenous TCR may impair the expression of transduced TCR, resulting in an insufficient antitumor capacity of modified T cells. Here, we report the development of immunotherapy using human lymphocytes transduced with a codon-optimized melanoma-associated antigen (MAGE)-A4 and HLA-A*2402-restricted TCR, which specifically downregulate endogenous TCR by small interfering RNA (si-TCR). We evaluated the efficacy of this immunotherapy in both NOD-SCID mice and uterine leiomyosarcoma patients. Our results revealed that transduced human lymphocytes exhibited high surface expression of the introduced tumor-specific TCR, enhanced cytotoxic activity against antigen-expressing tumor cells, and increased interferon-γ production by specific MAGE-A4 peptide stimulation. Retarded tumor growth was also observed in NOD-SCID mice inoculated with human tumor cell lines expressing both MAGE-A4 and HLA-A*2402. Furthermore, we report the successful management of a case of uterine leiomyosarcoma treated with MAGE-A4 si-TCR/HLA-A*2402 gene-modified T cells. Our results indicate that the TCR-modified T cell therapy is a promising novel strategy for cancer treatment.

## Background

In recent years, the development of immune checkpoint-based immunotherapy, such as PD-1/PD-L1 monoclonal antibodies, has been applied in a variety of tumors and shown good clinical results. However, immune checkpoint treatments are only effective in a small number of cancer patients, and thus new options and methods are needed^1^. Adoptive T cell therapy is a rapidly developing method of tumor immunotherapy. The principle is to introduce large numbers of in vitro amplified effector cells into the patient to produce a direct killing effect of the tumor cells. Two types of genetically modified specific T cell adoptive immunotherapies, chimeric antigen receptor T cells (CAR-T) and T cell receptor-engineered T cells (TCR-T), have been shown to be attractive for treating patients with malignancies^2,3^. CAR-T cell therapy produces significant clinical results in hematological tumors, but they are only specific for surface antigen and show limited applications in solid tumors^4,5^. In contrast, TCR-T cells recognize fragments of antigen as peptides bound to major histocompatibility complex (MHC) molecules and display good clinical effects in the treatment of solid tumors. Three NY-ESO-1/HLA-A2-specific TCR-T clinical trials in 2011 and 2015 showed that more than 50% of patients with synovial sarcoma, malignant melanoma, and multiple myeloma exhibited an objective clinical response, which encouraged the development of novel TCR-T cell immunotherapies^6–8^.

However, on-target adverse events in TCR gene therapy targeting melanocyte differentiation antigens, such as melanoma antigen (MART)-1 or gp100, etc., have been reported. Normal tissues such as the skin and brain, which express sequence-like antigens, show cross-recognition of TCR-T cells and severe destruction, particularly when using high-affinity TCR. Thus, optimal antigen selection is crucial for TCR-T treatment^9–11^.

Cancer/testis antigens are particularly attractive targets for immunotherapy because they are highly expressed on adult male germ cells or tumor cells, but not in normal adult tissues^12,13^. Melanoma-associated antigen (MAGE) family antigens are mainly expressed in many malignant tumors such as melanoma, but show low expression in normal tissues. Immunotherapy strategies for targeting these antigens have been well-studied^14,15^. High expression of MAGE-A4, a member of the MAGE-A family, was reported in ovarian cancer, melanoma, non-small cell lung cancer, and esophageal squamous cell carcinoma^16–18^. This suggests that TCR-T cell therapy targeting MAGE-A4 is feasible and promising treatment for malignant tumors.

During introduction of exogenous TCR into T cells, the presence of endogenous TCR leads to a mismatch between the two types of TCRs, which may result in recognition of unknown antigens expressed on normal tissues and cause tissue damage. There are several new developing strategies to minimize the risk of mixed TCR dimer and improve the expression of the introduced TCR, such as zinc-finger nucleases, meganucleases, non-viral genome targeting, TALEN technology, CRISPR technology, TCR inhibitory molecular (TIM) peptide as well as RNAi-mediated TCR knockdown^19–26^. Gene editing by zinc-finger nucleases is an appealing approach for shutting down TCR expression, but it takes a long time for T cell culture and also includes multiple sorting steps which was inconvenient for clinical applications^19^. The meganucleases only have a single domain which making it more difficult to be engineered. Moreover, the enzyme is too expensive to develop for clinical use^25^. For non-viral T cell genome targeting, the major barrier is the toxicity of the DNA at high concentrations^20^. TALEN technology has high precision and efficiency to target long gene sequences, but the plasmid construction is complicated and the off-target effect is also existed^23^. CRISPR technology is simple, low cost, easy to construct and very efficient. However, both cytotoxicity and off-target effects were reported^26^. Other exploratory approaches to prevent GVHD caused by allogen-reactive T cells such as the application of TIM peptide is an appealing strategy which has been designed in CAR-T treatment and the clinical trial is ongoing^27^. The use of RNAi-mediated gene editing is also fast, easy, reducing the number of experimental steps and saving time costs. But this method can’t completely remove the gene function. We and our collaborators chose the RNAi-mediated TCR editing by retrovirus transduction, which uses a one-step transduction protocol and have show the reduction of endogenous TCR efficiently. This approach meet our requirements for avoiding mismatches between exogenous and endogenous TCR and showed better T cell recognition and killing activity^28^.

In this study, we further evaluated the application of this RNAi-mediated TCR gene editing method in mice and human. We manufactured MAGE-A4-si-TCR gene-modified T cells using human peripheral blood mononuclear cells (PBMCs) and examined the in vitro activity of the T cells. Next, we introduced the gene-modified T cells into NOD-SCID mice bearing tumors with or without HLA-A2402 to assess its in vivo antitumor capacity. Moreover, we present clinical evidence of a case of uterine leiomyosarcoma adoptively transferred with MAGE-A4 si-TCR gene-modified T cells.

## Methods

### Cell lines

KE4 (MAGE-A4^+^ and HLA-A*2402^+^ human esophageal carcinoma), QG56 (MAGE-A4^+^ and HLA-A*2402^−^) human lung carcinoma), and T2A24 (human T, B hybridoma transfected with HLA-A2402 cDNA) cell lines were obtained from Takara Bio (Otsu, Japan) and all cells were cultured in RPMI-1640 media (Gibco, Grand Island, NY, USA) supplemented with 10% fetal bovine serum (Hyclone, Logan, UT, USA).

### Isolation and cultivation of peripheral blood mononuclear cells

Peripheral blood mononuclear cells (PBMCs) were derived from healthy donors (for in vitro and in vivo experiments) or patients (for adoptive immunotherapy) who signed informed consent. PBMCs isolated using Ficoll were cultured in GTT551 media (Takara Bio) supplemented with 1% autologous plasma, 0.2% human serum albumin (Shuyang Bio, Sichuan, China), and 1000 IU/mL interleukin-2 (Bailu Bio, Beijing, China).

### Preparation of gene-modified cells

The MAGE-A4 si-TCR retroviral vector was developed and provided by Takara Bio and shows high surface expression of the introduced tumor-specific TCR and reduced expression of endogenous TCRs^28^. Gene-modified cells were manufactured as described previously^28,29^. Briefly, PBMCs were stimulated with 30 ng/mL OKT-3 (eBioscience, San Diego, CA, USA) and 1000 IU/mL interleukin-2 prior to transduction, and then retroviral vector solutions were applied onto Retronectin (Takara Bio, Beijing) preloaded bags for 16 h at 4 °C. Stimulated cells were then cultured in vector-coated bags and expanded for 3–5 days. Control PBMCs (non-gene-modified cells, NGMCs) were cultured simultaneously.

### Flow cytometric analysis and tetramer assays

Fluorescein isothiocyanate (FITC)-conjugated antihuman CD2, CD3, CD8, and CD45 and phycoerythrin (PE)-conjugated antihuman CD4, CD14, CD19, and CD56 monoclonal antibodies were all from BD Biosciences (Franklin Lakes, NJ, USA) and used to detect the immunophenotypes of transduced T cells. PE-conjugated MAGE-A4_143–151_/HLA-A*2402 tetramer (from TCMetrix, Epalinges, Switzerland) was also used to detect TCR expression in gene-modified cells and PBMCs from patient’s blood. The results for tetramer analysis were defined as the percentage of tetramer positive cells in CD8 positive cells. Data were acquired with a FACS CantoII flow cytometer (BD Biosciences) and analyzed using FACSDiva and FlowJo software (Ashland, OR, USA).

### Cell killing experiment

50–100 thousand CFSE (Thermo Fisher Scientific, USA) labeled target cells per well were seeded in triplicate in 12-well plates for 1 days, then were cocultured with effector cells at an effector/target ratio of 10:1. Four hours later, all cells were collected and stained with NIR-Zombie dye (Biolegend, USA) for cell viability assay. The results were analyzed by flow cytometry and the percentage of dead cells was considered as the cell killing rate.

### ELISPOT assay

Human ELISPOT assays (Dakewe, Nanshan, China) were used to detect the secretion of IFN-γ after specific peptide stimulation. Briefly, cells with or without MAGE-A4 peptide were seeded into a 96-well plate that had been precoated with a capture antibody specific for human IFN-γ. After 24 h of incubation in a humidified 37 °C, 5% CO_2_ incubator, the wells were washed and biotinylated detection antibody specific for human IFN-γ was added to the wells. Unbound biotinylated antibody was washed away and horseradish peroxidase-conjugated antibody was added. Following four washes to remove unbound enzyme, substrate solution was added. A colored precipitate formed and appeared as spots at the sites of cytokine localization. The spots were counted by computer-assisted image analysis (ImmunoSpot Series 3 Analyzer: CTL, Shaker Heights, OH). PMA and Ionomycin (Sigma-Aldrich) were used to stimulate the cells as positive control.

### IFN-γ assay

Target cells (T2A24) were pulsed with 4 μg/μL MAGE-A4_143–151_ peptide for 3 h and then were cocultured with effector cells at an effector/target ratio of 1:1. Two hours later, GolgiPlug (BD Biosciences) was added to the samples at 1 μL/mL and then the cells were further incubated for another 20 h. For staining, the cells were first stained with FITC-conjugated anti-CD8 and then permeabilizated using a IntraPrep kit (Beckman Coulter, Brea, CA, USA) following the manufacturer’s instructions. The cells were further stained intracellularly with PE-conjugated anti-IFN-γ (Beckman Coulter) and analyzed by flow cytometric analysis.

### Mice

Six to eight-week-old female NOD-SCID mice (Beijing Vital River Laboratory Animal Technology Co., Ltd., Beijing, China) were used in this study. All experimental protocols were approved by the Ethics Review Committee for Animal Experimentation (Tianjin Medical University Cancer Institute and Hospital).

### Tumor inoculation and adoptive cell transfer

KE4 tumor cells (1 × 10^7^ in 0.2 mL PBS) and QG56 tumor cells (1 × 10^6^ in 0.2 mL PBS) were subcutaneously injected into the right flank of NOD-SCID mice. Mice were randomly divided into three groups and administered different treatments through the tail vein: normal saline group (NS), NGMC group (1 × 10^8^ cells), and GMC (1 × 10^8^ cells) group. The condition of mice was monitored every day and the tumor size was measured with calipers via perpendicular diameters twice per week. Safety was evaluated by observing the general status, response, and body weight of mice and efficacy was evaluated by observing the tumor growth of KE4 and QG56.

### Detection of MAGE-A4 antigen expression

Expression of MAGE-A4 was assessed by immunohistochemistry (IHC) using the monoclonal antibodies MCV-1 and MCV-4, which were kindly provided by Mie University, Japan^29^. MCV-1 and MCV-4 recognize amino acids 255–277 and 71–95 of MAGE-A4. MCV-1 reacted with MAGE-A2, MAGE-A4, and MAGE-A12, while MCV-4 covered MAGE-A1 and MAGE-A4, respectively. The immunoreactions were separately evaluated by two pathologists. Tissue samples with 5% positive or more for both MCV-1 and MCV-4 staining were judged as MAGE-A4 positive.

### Treatment protocol

The patient was both MAGE-A4 and HLA-A2402 positive and administered a preconditioning regimen (Cyclophosphamide 10 mg/kg for 2 days and Fludarabine 25 mg/m^2^ for 2 days), followed by the manufactured gene-modified lymphocytes intravenously on days 0 and 14. Interleukin-2 (1 million/m^2^ for 2 days and 2 million/m^2^ for 5 days) infusion was conducted for 7 days. On days 21 and 28, the patient was subcutaneously administered 300 mg of MAGE-A4 peptide (NYKRCFPVI; PolyPeptide Laboratories, Torrance, CA, USA) emulsified with incomplete Freund adjuvant (Montanide ISA-51VG; SEPPIC, Paris, France). On days 56 and after, safety and clinical responses were assessed, and the patient was followed up until March, 2019. This study was approved by the Research Ethics Committee of Tianjin Medical University Cancer Institute and Hospital, China. Written informed consents were obtained from the patient before participating in the study.

### Measurement of proviral copy number for cell kinetics analysis

DNAs were isolated from patient’s blood using the DNA extraction kit (Qiagen). Primers for proviral DNA sequence (retroviral packaging signal region, existing in TCR transduced cells) and human IFNg DNA (genes of whole T cells) were from Provirus Copy Number Detection Primer Set (6167, Takara Bio Inc.). qPCR assay were performed using Cycleave PCR Core Kit (CY501, Takara Bio Inc.). The DNA samples were amplified by 50 cycles of 3-step PCR reactions. Serially diluted DNA control template for provirus and IFNg were amplified and the standard curve was generated. Each DNA concentration of IFNg or proviral vector for MAGE-A4 si-TCR expression was calculated from the standard curve. The copy number was represented as the ratio of proviral DNA and IFNg DNA values.

### Statistical analysis

Data were analyzed using GraphPad Prism version 5 (GraphPad Software, San Diego, CA). Differences between cell and tumor growth were determined by repeated measures ANOVA. The Mann–Whitney *U*-test and Student’s *t*-test were used for parameter comparison between two subject groups. A *P*-value less than 0.05 was considered statistically significant.

## Results

### Manufacture of lymphocytes transduced with MAGE-A4 si-TCR

**Fig. 1:**
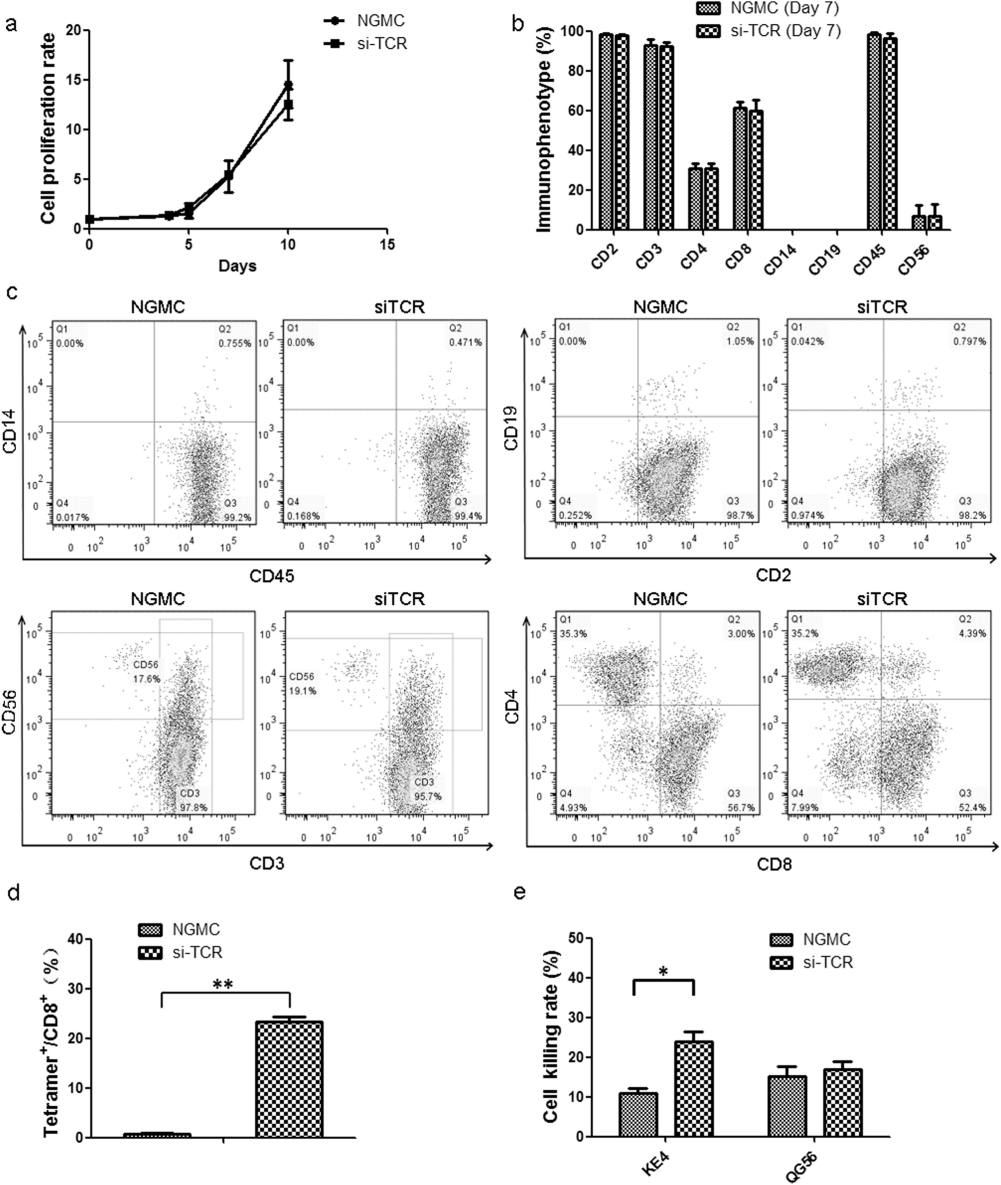
Growth curve, immunophenotype, tetramer, and cell killing analysis of manufactured melanoma-associated antigen (MAGE)-A4 si-TCR gene-modified cells. **a** Transduction of si-TCR in human lymphocytes. Peripheral blood mononuclear cells from healthy donors were stimulated with anti-CD3 mAb and interleukin-2. Cells were cultured with or without retroviral transduction. Cell growth rate of si-TCR T cells and NGMC cells were monitored and the growth curve is shown. **b** Immunophenotype of si-TCR GMCs and NGMCs were detected by flow cytometric analysis and the histogram is shown. **c** Representative staining for gene-modified and unmodified cells with antibodies for cell surface markers. **d** GMCs and NGMCs were stained with MAGE-A4 tetramer and anti-CD8 mAb, percentage of tetramer positive cells among CD8^+^ cells is indicated. **e** Cell killing assay was performed in GMCs and NGMCs cocultured with KE4 and QG56 cell lines and the results were shown. All experiments were done at least from 3 donors’ PBMC and the representative data was shown. Repeated measures ANOVA was used for cell growth rate analysis and Mann–Whitney *U*-test was used for others. Data represent mean ± SEM, *P < 0.05, **P < 0.01

The generation of retroviral vectors encoding both small interfering RNA constructs that specifically downregulate endogenous TCR and a codon-optimized, small interfering RNA-resistant TCR specific for the human tumor antigens MAGE-A4 (named as si-TCR) were described previously^28^. Lymphocytes from at least three different healthy donors were isolated and transduced with retrovirus carrying MAGE-A4 si-TCR. The gene-modified T cells (GMCs) were manufactured on a large scale in vitro and the cell growth curve was drawn according to the cell number account results. There was no significant difference in cell growth between GMCs and non-gene modified cells (NGMCs), indicating that the retrovirus transduction method did not influence the cells (Fig. 1a). Flow cytometry was performed to detect the immunophenotype of the modified cells, evaluated as the expression of cell surface markers, including CD3, CD4, CD8, CD2, CD19, CD56, CD14, and CD45. The results revealed that the immunophenotype of GMCs and NGMCs were similar and mainly exhibited the T cell phenotype, particularly CD8^+^ killer cells (Fig. 1b, c).

To evaluate the expression of introduced codon-optimized TCR, MAGE-A4_143–151_/HLA-A*2402 tetramer and antihuman CD8 mAb were stained for flow cytometric analysis. The results showed cell surface MAGE-A4–specific TCR expression was much higher in si-TCR cells than in control NGMCs (Fig. 1d, *P* < 0.01). To further validate the cytotoxic ability of GMCs towards cell lines with and without MAGE-A4/HLA-A*2402, KE4 (MAGE-A4^+^ and HLA-A*2402^+^) and QG56 (MAGE-A4^+^ and HLA-A*2402^−^) cells were used for the cell killing assay. After cocultured for 4 h, the cell killing effect observed on KE4 was significant increased in si-TCR cells than in NGMCs, while there was no significant difference between the killing effect on QG56 (Fig. 1e, *P* < 0.05). All experiments were performed at least for three independent times and the representative data was shown.

### Si-TCR gene-modified cells produced more interferon-γ after specific MAGE-A4 peptide stimulation in vitro

**Fig. 2:**
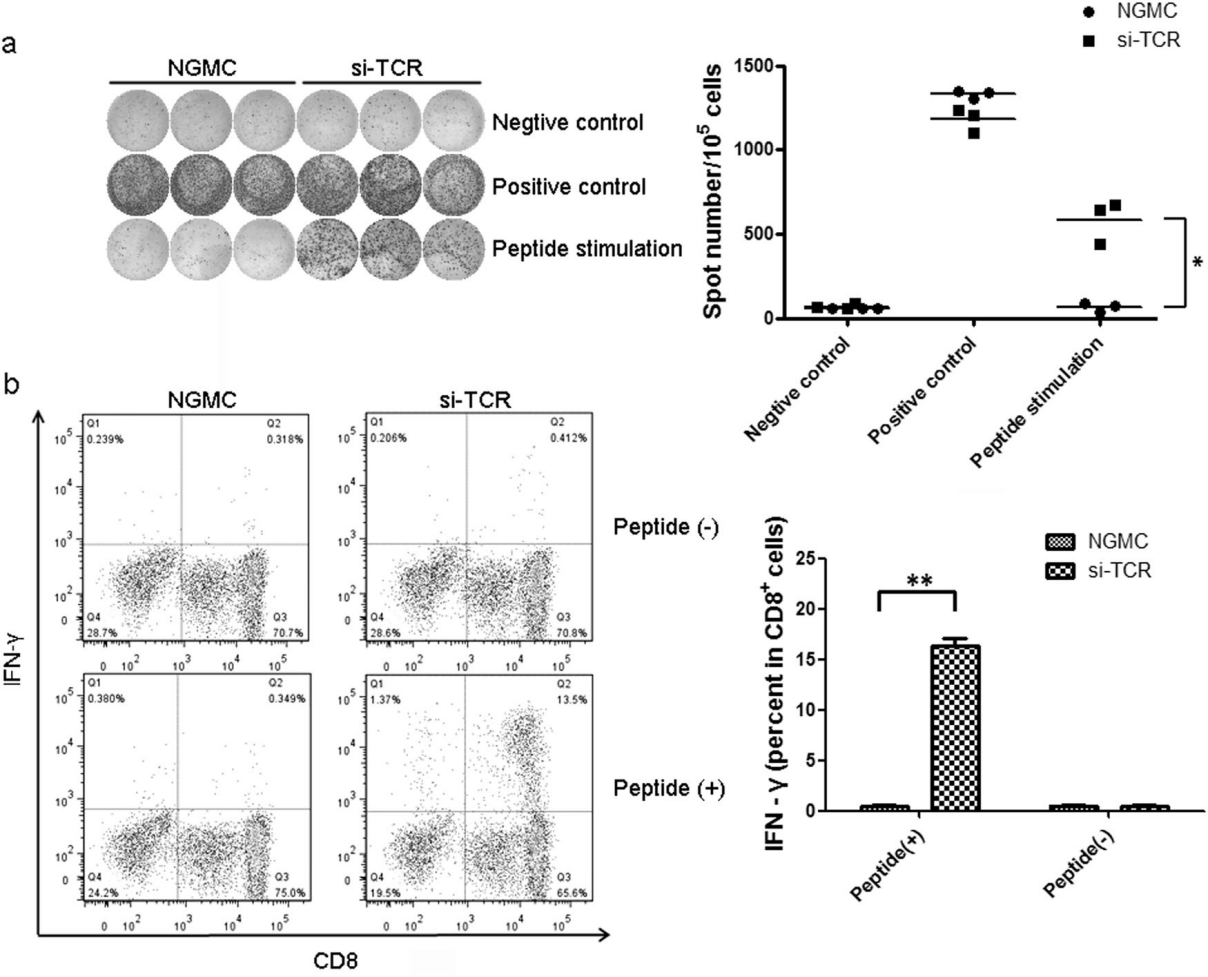
In vitro functional analysis of MAGE-A4 si-TCR gene-modified cells. **a** si-TCR GMCs and NGMCs were stimulated in vitro with MAGE-A4 peptide, and then subjected to ELISPOT assays. PMA/Ionomycin stimulation was used as a positive control. The right panel shows the scatter plot. **b** si-TCR gene-modified cells and unmodified cells were stimulated with T2A24 cells pulsed with the MAGE-A4_143–151_ peptide and subjected to an IFN-γ assay by flow cytometry. Representative staining is shown in the left panel. Mann–Whitney *U*-test was used to determine the significance of differences between two groups, Data represent mean ± SEM, *P < 0.05, **P < 0.01

To further investigate the function of GMCs, an ELISPOT interferon (IFN)-γ assay was conducted after MAGE-A4_143–151_ peptide stimulation. As a positive control, we stimulated both GMCs and NGMCs with PMA/Ionomycin. The number of spots developed in GMCs after peptide-specific stimulation was significantly higher than that in NGMCs following the same stimulation (Fig. 2a, left panel). A scatter plot is shown in the right panel (*P* < 0.05). Moreover, to verify that the GMCs react specifically with HLA-A*2402-restricted MAGE-A4, we measured intracellular IFN-γ secretion by flow cytometry, which also reflected the GMC gene transduction efficiency (ratio of IFN-γ positive in CD8^+^ cells). The peptide-loaded T2-A*2402 cells (T2A24) were used as target cells. The results revealed that GMC cells produced IFN-γ specifically after MAGE-A4 peptide stimulation (Fig. 2b left panel); the histogram is shown in the right panel (*P* < 0.01). At least three independent experiments were done and the representative data was shown in the Figure. These results indicate that si-TCR GMCs released IFN-γ against cells expressing both MAGE-A4 and HLA-A*2402.

### Adoptive transfer of si-TCR gene-modified cells suppressed tumor growth in NOD-SCID mice inoculated with human tumor cell lines expressing both MAGE-A4 and HLA-A*2402

**Fig. 3:**
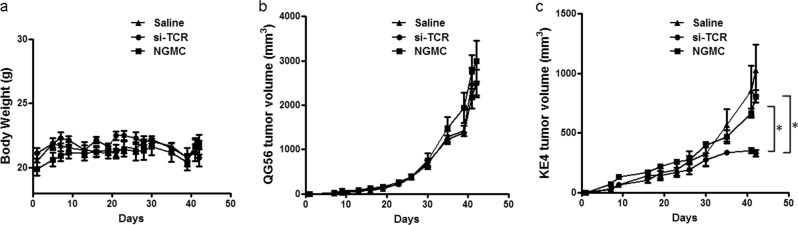
Adoptive transfer of si-TCR gene-modified cells inhibits human tumor growth in NOD-SCID mice. **a** NOD-SCID mice (*n* = 5 per group) were subcutaneously inoculated with KE4 tumor cells (1 × 10^7^ in 0.2 mL PBS) and QG56 tumor cells (1 × 10^6^ in 0.2 mL PBS) and then intravenously administered with saline, GMCs, and NGMCs (produced from healthy donors). At least three independent experiments were done and the representative data was shown. The body weight is shown as the mean ± SEM. **b**, **c** The tumor size in each group (b for QG56 tumor and c for KE4 tumor) was measured and is shown as the average ± SEM. Repeated measures ANOVA was used for analysis. *P < 0.05 (si-TCR group vs. Saline group and si-TCR group *vs*. NGMC group)

To evaluate the safety and efficacy of the gene-modified cells in vivo, adoptive cell therapy of si-TCR gene-modified cells (manufactured from healthy donors) into NOD-SCID mice was conducted to confirm the antitumor response. Eight-week-old female NOD-SCID immunodeficient mice were inoculated subcutaneously with KE4 (1 × 10^7^ cells/mouse) and QG56 (1 × 10^6^ cells/mouse) into the right flank of mice. After inoculation with tumor cells, mice were randomly divided into three groups and administered three different treatments: normal saline group (NS), NGMC group, and si-TCR GMC group. We evaluated the safety of these treatments by observing the general status, response, and weight of the mice. Anti-tumor effectiveness was evaluated by measuring the tumor growth of KE4 and QG56. At least three independent experiments were performed and the representative data was shown. The results revealed no significant side effects or toxicity in the three experiment groups (Fig. 3a). Compared to the NS group and NGMC group, si-TCR GMCs transferred into KE4 tumor-bearing mice inhibited tumor growth specifically, while this effect was not observed in QG56 tumor-bearing mice (Fig. 3b, c, *P* < 0.05), indicating that administration of si-TCR gene-modified lymphocytes inhibited human tumor growth in NOD-SCID mice in a specific manner for MAGE-A4^+^HLA-A*2402^+^ KE4 tumors.

### Uterine leiomyosarcoma patient treated with MAGE-A4 si-TCR/HLA-A*2402 gene-modified T cells after chemotherapy obtained a stable status for 41 months; a pilot trial

**Fig. 4:**
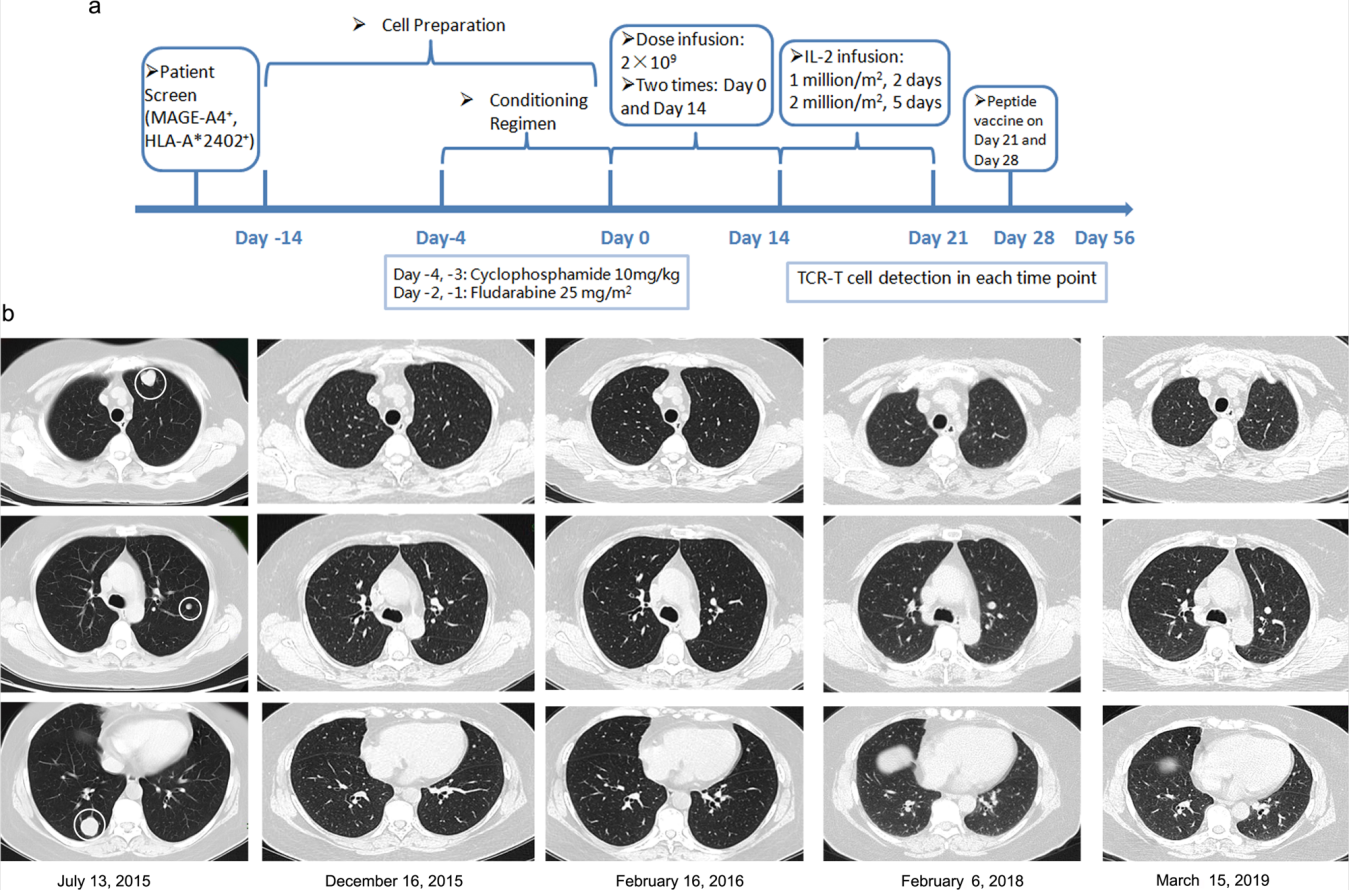
Treatment protocol and comparison of the patient’s CT scan images before and after MAGE-A4 si-TCR gene-modified cell therapy. **a** The treatment protocol for patient was shown. **b** Tumor masses were observed in the left lung hilum and mediastinal lymph nodes of the patient (white circles) before initiation of chemotherapy (July, 13, 2015). The masses significantly reduced in size on CT obtained 60 days after chemotherapy (December 16, 2015). TCR gene-modified cell treatment was administered after chemotherapy and the CT scanning was performed (February 16, 2016). After TCR-T cell treatment, CT scanning was conducted every 6 month, which revealed the stable maintenance of the CR status for more than 3 years (March 15, 2019)

To evaluate the clinical efficacy of MAGE-A4 si-TCR GMCs, we conducted a pilot trial in a HLA-A*2402 uterine leiomyosarcoma patient. MAGE-A4 expression was assessed by immunohistochemistry (IHC) and the results were judged as positive (Fig. S1). The female patient was diagnosed with uterine leiomyosarcoma on August 14, 2014 (Pathology: II A) and underwent operation. Three cycles of adjuvant chemotherapy with L-OHP + IFO + EPI were performed from September 18, 2014 to November 11, 2014 and pelvic field-assisted radiotherapy was performed on December 2014. After 5 months, the computed tomography (CT) scan results revealed double lung metastasis (pathology: malignant tumor, sarcoma) and 1 round of chemotherapy with bevacizumab + CBP + DTIC was performed on June 15, 2015. One month later, CT scanning showed disease progression, and 4 rounds of second-line chemotherapy with ENDOSTAR + DOX + GEM were performed from July 2015 to October 2015. After chemotherapy, the treatment efficacy showed a complete response (CR). Because the tumor burden was at a lower level, we administered TCR gene-modified cell treatment. PBMCs from patient were separated by apheresis. Cells were stimualted with IL2, anti-CD3 and then transduced with the si-TCR vector. After 7–10 days culture, cells were harvested and then frozen until use. Quality detection of si-TCR T cell products were performed as described above (Fig. S2). The treatment schema was shown in Fig. 4a, as depicted, MAGE-A4 si-TCR cells were infused to the patient for 1 cycle (twice) according to the clinical protocol on Day 0 and Day 14 after low-dose lymphodepletion (Cyclophosphamide 10 mg/kg for 2 days and Fludarabine 25 mg/m^2^ for 2 days). Interleukin-2 (1 million/m^2^ for 2 days and 2 million/m^2^ for 5 days) infusion was conducted for 7 days after the second infusion. On Day 21 and 28, the patient was subcutaneously administered 300 mg of MAGE-A4 peptide. On days 56 and after, safety and clinical responses were assessed. The treatment was well-tolerated with no treatment-related morbidity, life-threatening complications, or side effects. The patient has been followed-up until March, 2019, that is for more than 3 years, the efficacy remains CR (Fig. 4b). This evidence supports the application of si-TCR GMCs in clinical treatment.

**Fig. 5:**
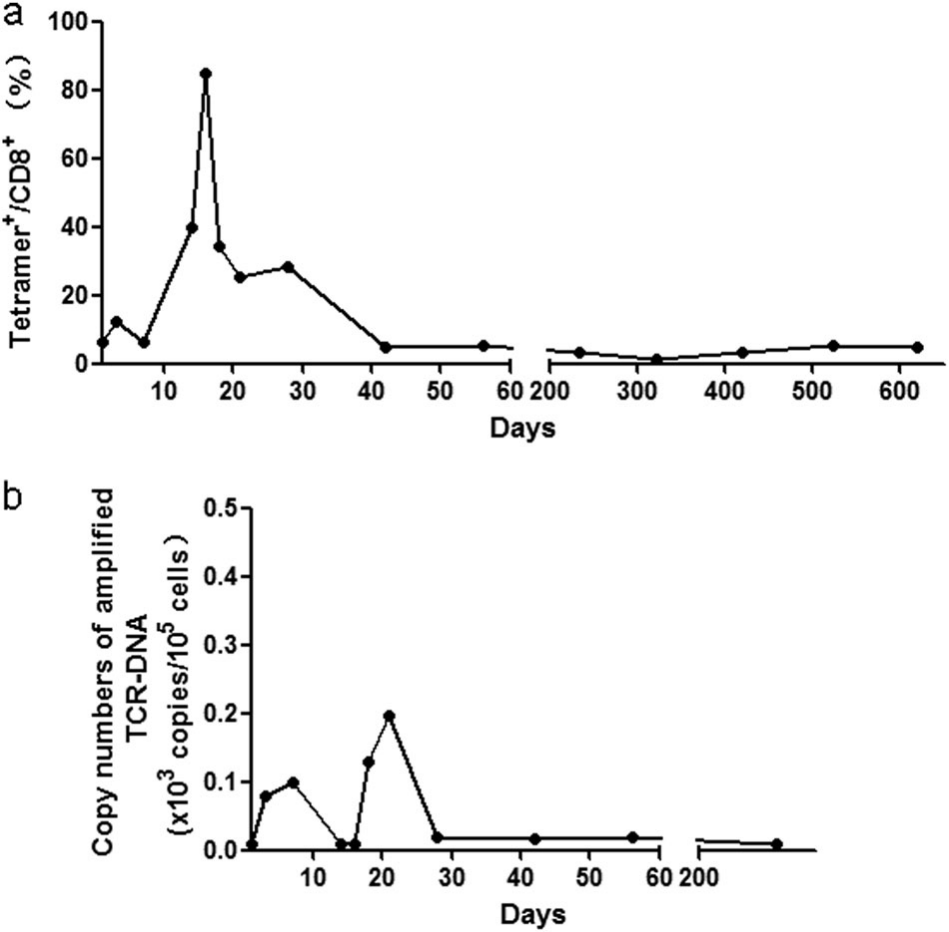
Tetramer and copy number analysis of patient’s blood. **a** PBMCs were collected at different time points after the adoptive transfer of si-TCR T cells and directly stained with MAGE-A4 peptide/HLA-A*2402 tetramer. **b** Blood genome DNA was extracted at different time points and applied to copy number assays

In general, patients with sarcoma do not receive any other treatments will relapse soon after chemotherapy. We speculate that the reason for our patient to maintain long-term CR might be due to the si-TCR immunotherapy. Hence we detected both the MAGE-A4 tetramer and copy number in patient’s blood to investigate the long-term persistence of transduced T cells. Over more than 600 days observation, the tetramer^+^CD8^+^ T cells were detected and the TCR transgene copies were also observed until Day 233 (Fig. 5a, b). Both these two indicators have a peak value between Day 20 and Day 30, suggesting the effect of MAGE-A4 peptide vaccination. These results indicated the long-term persistence of si-TCR T cells in the patient.

## Discussion

In this study, we manufactured human lymphocytes which simultaneously express a codon-optimized MAGE-A4 TCR and siRNAs to silence endogenous TCR. The modified T cells showed high surface expression of introduced tumor-specific TCR and enhanced antigen-specific cytotoxicity for target cells. NOD-SCID mice inoculated with human tumor cell lines expressing both MAGE-A4 and HLA-A*2402 exhibited decreased tumor growth after si-TCR T cell treatment. Clinical evidence also showed that the patient was well-tolerated after si-TCR T cell therapy and maintained a stable status.

TCR-T cell therapy using retrovirus as a vector was first implemented in melanoma more than 10 years ago^10^. Morgan et al. reported a phase I clinical trial of patients with malignant melanoma using TCR-T cells specific for the melanoma antigen-1. No serious adverse events were observed, and partial clinical responses were reported. Subsequently clinical trials demonstrated that TCR-T cell therapy has shown good clinical effects in the treatment of solid tumors such as multiple myeloma, synovial sarcoma, and melanoma^6,8,30,31^. On February 9, 2016, the FDA granted breakthrough therapy designation for the Adaptimmune’s affinity enhanced T-cell therapy targeting NY-ESO-1 in synovial sarcoma. However, one problem associated with introduction of exogenous TCR is the mismatch with endogenous TCR, which leads to T cell recognition of normal tissues expressing unknown antigens to cause tissue damage^11,32–34^. In our study, the introduction of endogenous small interfering RNA allowed T cells to express TCRs of specific epitopes while interfering with the expression of inherent TCR. This design avoids the mismatch of exogenous and endogenous TCR and enhances T cell recognition and killing activity.

High expression of MAGE-A4 was reported in several solid tumors such as ovarian cancer, melanoma, non-small cell lung cancer, and esophageal squamous cell carcinoma^17,35^. In this study, we used MAGE-A4 as the target antigen and developed a vector carrying both the MAGE-A4 tumor antigen TCR gene and small interfering RNA vector (MAGE-A4 si-TCR). We manufactured large numbers of gene-modified T cells and performed in vitro and in vivo detection of anti-tumor activity. Compared to non-transduced cells, genetically modified T cells showed no significant differences in cell morphology or growth rate, indicating that the transduction did not affect the growth characteristics of the cells. Simultaneously, the surface markers of cells were detected by flow cytometry. Both genetically modified and un-modified cells showed phenotypes of T lymphocytes, which were mainly CD8^+^ cytotoxic T cells. Tetramer assay showed the cell surface MAGE-A4-specific TCR expression was much higher in si-TCR cells than in control NGMCs and cytotoxic assay indicated the specific cell killing effect toward MAGE-A4^+^, HLA-A*2402^+^ cell lines. Moreover, functional analysis showed that the genetically modified cells produced more IFN-γ following stimulation of specific MAGE-A4 peptide. For in vivo experiment, we observed the general state and response of NOD-SCID mice infused with si-TCR T cells, no adverse reactions or side effects were detected. After infusion, the growth of KE4 tumors (MAGE-A4^+^, HLA-A*2402^+^) was specifically inhibited, while the tumor size of QG56 tumors (MAGE-A4^+^, HLA-A*2402^−^) was not changed, indicating that the modified T cells specifically killed their HLA matching KE4 tumors.

Interestingly, we found clinical evidence of a uterine leiomyosarcoma patient who showed a long-lasting CR after chemotherapy following TCR-T cell treatment. Although the patient had a CR following the last round of chemotherapy, it is very rare to maintain CR for more than 3 years without any other treatments. Our results for tetramer and copy number detection indicated the persistence of si-TCR cells in the patient blood. All these findings enable us to believe that the si-TCR immunotherapy is the main reason for the patient to maintaining long-term CR.

In summary, we successfully manufactured MAGE-A4 si-TCR gene-modified T cells and both in vitro and in vivo tests indicated its specific activity toward MAGE-A4 and HLA-A*2402 positive tumor cells. Clinical evidence for a patient also suggested that si-TCR T cell therapy may attribute to maintaining a CR status. Our data suggest the adoptive cell therapy with human lymphocytes engineered to express MAGE-A4 si-TCR is a promising strategy to treat patients with MAGE-A4 expressing tumors. Further studies are being conducted at our institution to validate the clinical application of TCR-T therapy.

## Supplementary information

Figure S1

Figure S2

Supplementary figure legends
