# Supplementary figures and images for: T-cell receptor gene therapy targeting melanoma-associated antigen-A4 by silencing of endogenous TCR inhibits tumor growth in mice and human
Source: Cell Death Dis. 2019 Jun 17;10(7):475. doi: 10.1038/s41419-019-1717-8 (PMC6572850; doi:10.1038/s41419-019-1717-8)

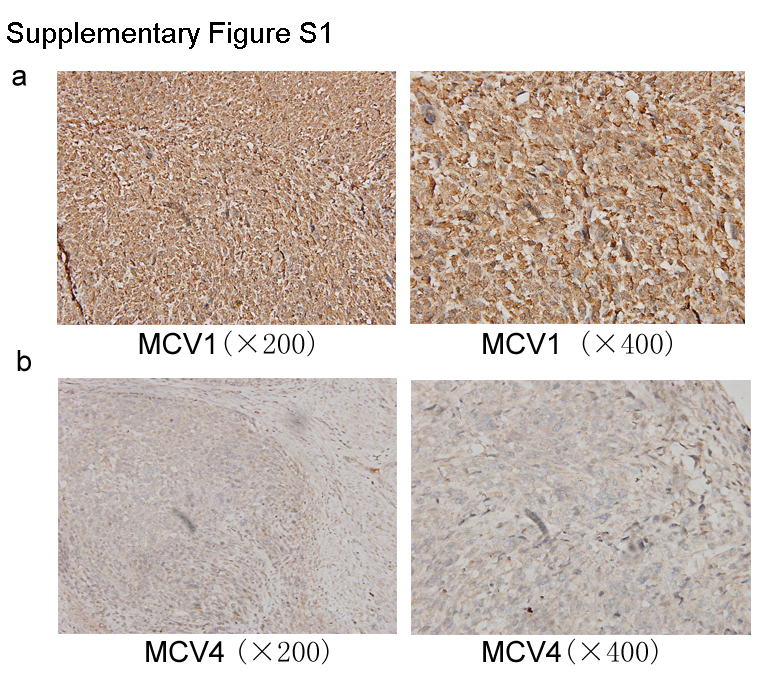

Supplement: Supplementary file 1 — Figure S1 [file 41419_2019_1717_MOESM1_ESM.tif]

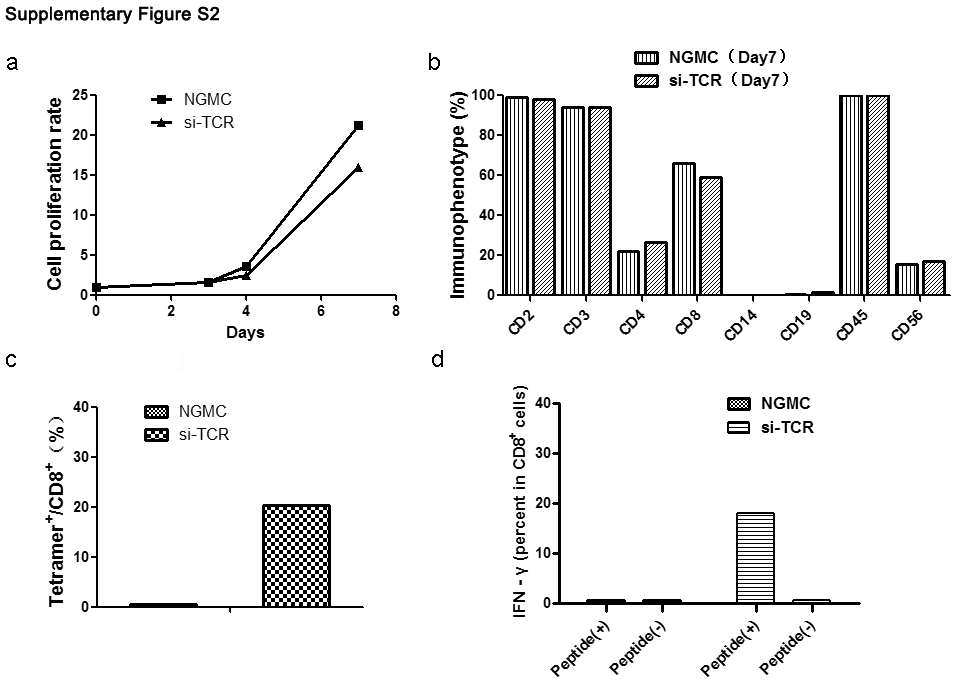

Supplement: Supplementary file 2 — Figure S2 [file 41419_2019_1717_MOESM2_ESM.tif]
