# Supplementary material for: T-cell receptor gene therapy targeting melanoma-associated antigen-A4 by silencing of endogenous TCR inhibits tumor growth in mice and human
Source: Cell Death Dis. 2019 Jun 17;10(7):475. doi: 10.1038/s41419-019-1717-8 (PMC6572850; doi:10.1038/s41419-019-1717-8)
Supplement: Supplementary file 3 — Supplementary figure legends [file 41419_2019_1717_MOESM3_ESM.docx]

**Fig. S1** **IHC analysis of MAGE-A4 in patient’s paraffin-embedded tumor tissues.** MCV-1 and MCV-4 recognize different amino acids of MAGE-A4 and tissue samples positive for both MCV-1 and MCV-4 staining were judged as MAGE-A4 positive. Pictures for ×200 and ×400 were shown.

**Fig. S2** **Quality detection results of si-TCR T cell products manufactured from patient.** Data of growth curve **a**, immunophenotype **b**, tetramer **c** and IFN-γ analysis **d** for gene-modified cells were shown.
